# The Comparison of Inflammatory Cytokines between Spinal and General Anesthesia following Changes in Ischemic Reperfusion due to Tourniquet during Lower Limb Surgery

**DOI:** 10.1155/2021/2027421

**Published:** 2021-09-09

**Authors:** Mahmoud Ganjifard, Samaneh Kouzegaran, Reza Abdi, Mohsen Naseri, Elahe Allahyari, Amir Sabertanha, Batool Zeinali

**Affiliations:** ^1^Department of Anesthesia, Faculty of Medicine, Birjand University of Medical Sciences, Birjand, Iran; ^2^Clinical Immunology and Allergy, Faculty of Medicine, Birjand University of Medical Sciences, Birjand, Iran; ^3^Department of Orthopedics, School of Medicine, Ghaem Hospital, Mashhad University of Medical Sciences, Mashhad, Iran; ^4^Cellular and Molecular Research Center, Birjand University of Medical Sciences, Birjand, Iran; ^5^Social Determinants of Health Research Center, Department of Epidemiology and Biostatistics, Faculty of Health, Birjand University of Medical Sciences, Birjand, Iran

## Abstract

**Methods:**

In this randomized controlled clinical trial, 34 patients with lower limb surgery admitted at the orthopedic ward of Imam Reza Hospital, Birjand, Iran, were selected by the available sampling method. They were randomly divided into two groups as follows: general anesthesia (*n* = 17) and spinal anesthesia (*n* = 17). Venous blood samples were taken from the patients of both groups at baseline (before the use of tourniquet) and 12 and 24 hours after reperfusion. Interleukin-6 (IL-6), tumor necrotizing factor-*α* (TNF-*α*), high-sensitivity C-reactive protein (hs-CRP), and ferritin were measured and recorded. The data were analyzed using independent *t*-test, chi-square, and repeated measure at the significant level of 0.05.

**Results:**

The results showed that hs-CRP and IL-6 significantly increased during the study (*p* < 0.001); however, the mean changes of TNF-*α* and ferritin were not significant during the study. Moreover, none of the inflammatory cytokines indicated significant differences between these two study groups (*p* < 0.05).

**Conclusion:**

According to the results, the use of tourniquet can lead to inflammation, and the inflammation is similar in both groups.

## 1. Introduction

The use of tourniquet is an integral part in various methods of organ surgery, which is more commonly used in orthopedic surgery to reduce blood loss and improve vision in the surgical field [1]. Although new tourniquets have been designed in terms of the principles to reduce the incidence of possible complications, their usage is still associated with specific local complications such as neurological paralysis, vascular injury, and compartment syndrome [2, 3]. In addition, ischemia-reperfusion due to the tourniquet, which can lead to ischemia-reperfusion injury (IRI), is an important complication following surgery [4, 5]. IRI phenomenon leads to specific hemodynamic and metabolic responses, and the hallmarks are systemic and local inflammatory reactions. Although ischemic reperfusion is necessary to prevent irreversible cellular injury, reperfusion itself can more increase tissue injury compared to ischemia alone [6].

General and spinal anesthesia are widely used in orthopedic surgery resulting in varying amounts of oxygen to the patient as well as systemic inflammatory responses [7]. The results of a randomized controlled experiment showed that spinal anesthesia (SA) significantly reduced postoperative vascular complications (deep vein thrombosis, pulmonary embolism, and heart attack), bleeding, and infection and caused ventilation/perfusion mismatch and failure [8]. It is assumed that the improvement in postoperative death is due to a change in the “stress response” induced during the surgery using spinal anesthesia [9]. Interleukin-6 (IL-6) levels, as a valid index for systemic inflammatory response, are related to the extent of reperfusion injury [10, 11]. It has been reported that elevated IL-6 levels are delayed by neutrophil apoptosis and are associated with modulating the epithelial permeability in the air paths [12, 13]. In patients undergoing lower limb reperfusion/ischemia in aortic surgery, IL-6 and IL-8 levels have been shown to be associated with increased protein permeability in the lungs and also significant impairment of gas exchange [14, 15]. However, recent studies have shown that plasma malondialdehyde (MDA), IL-6, and IL-8 levels can rise, compared to baseline, between two and 24 hours after the tourniquet opens, and in patients undergoing very minor surgery the tourniquet opens after 6 hours; plasma MDA, IL-6, and IL-8 levels reached baseline; however in few studies, the effect of spinal and general anesthesia on systemic inflammatory responses was compared among patients with IRI. Therefore, methods reducing the severity of this response are useful in certain high-risk individuals. Along with interleukins, several markers such as high sensitivity C-reactive protein (hs-CRP) and ferritin have also been identified as enhancing markers in response to inflammation, though the changes following ischemia-reperfusion injury have been studied in very few studies [16, 17]. According to the studies done before, time-dependent closure of the tourniquet leads to ischemic changes in distal tissues to the tourniquet closure site [18–20]. After opening the tourniquet and reperfusion, ischemic products cause systemic inflammatory changes and inflammatory cytokines release. The release of these cytokines as well as the increase of body temperature can delay the patient wound healing, cause morbidity, and increase hospitalization [21]. Since spinal anesthesia reduces the discharge of catecholamines from the nerve terminal in the lower limbs, and regarding the role of catecholamines in cellular metabolism processes [22], it seems that preclosure tourniquet in spinal anesthesia can play an important role in reducing cellular metabolism processes and result in a preconditioning against tourniquet-induced ischemia [23]. According to the performed studies, the use of anesthetic medicines also slightly reduces cell metabolism and can reduce the ischemic changes induced by tourniquet [24, 25]. Therefore, this study aimed to compare the effects of general and spinal anesthesia on inflammatory cytokines (interleukin-6 and tumor necrotizing factor-*α* (TNF-*α*)) and acute phase proteins including hs-CRP and ferritin induced by changes in ischemic reperfusion, due to pneumatic tourniquet during lower limb surgery.

## 2. Patients and Method

In this randomized controlled clinical trial, 34 patients undergoing lower limb surgery hospitalized in orthopedic ward of Imam Reza Hospital in Birjand, Iran, from December 2018 to July 2019 were selected by available sampling method. The inclusion criteria were as follows: (1) Patients with American Society of Anesthesiologists score of 1–2; (2) age ranging from 25 to 65 years; (3) lower limb surgery by the use of tourniquet; (4) use of tourniquet on one of the lower limbs; (5) absence of any vascular, respiratory, or kidney failure, liver function disturbance, and diabetic disease; (6) using no antioxidant or anti-inflammatory drug. The exclusion criteria of the study were as follows: (1) duration of surgery lower than 60 min and more than 90 min; (2) need of second surgery; (3) patients unwillingness to participate in the study at any stage of the study; (4) the death of patients. The current study was ethically approved by Medical Ethics Committee of Birjand University of Medical Sciences (ir.bums.rec.1396.178). The study was performed in terms of the principles of the Declaration of Helinski. Eligible patients were selected and after explaining the purpose of the study and assuring patients that the information will remain confidential and also after obtaining informed consent, the patients were included in the study. Since some patients were discharged from the hospital prior to taking supplementary samples, measurement of the studied factors was performed in 34 samples. Qualified patients (34 patients) were randomly divided into two groups as follows: general anesthesia (*n* = 17) and spinal anesthesia (*n* = 17). This sample size had acceptable power according to *α*: 0.05 and *β*: 0.8.

In both groups, intravenous cannulation with catheter 18 was performed on the peripheral veins of the upper limbs, and then 500 mL of normal saline was injected. In the general anesthesia group, anesthesia was induced with 1 mg of midazolam (30 to 50 mg/kg), 2 *μ*g/kg of fentanyl, 0.5 mg/kg of atracurium, and 2 mg/kg of propofol. For maintenance of anesthesia, propofol at a dose of 100 *μ*g/kg was used per minute, which can be increased to 200 *μ*g/kg as needed. Atracurium was repeated every 30 minutes at one-quarter of the initial dose. In the case of hypotension, 500 mL of normal saline was first injected, 10 mg of ephedrine was intravenously injected if hypotension was more than 20% baseline, and blood pressure was precisely controlled with fluid therapy and inotropes. In the spinal anesthesia group, the patients were in the sitting position and we performed prep and drap of the third and fourth lumbar midline with Quincke needle number 25 treated with 2.5 mL bupivacaine 0.5% spinal anesthesia. The sensory level was checked using a needle every two minutes. When the sensory level reached the 12th lumbar vertebra, the bed position was returned to neutral, and then the surgical team was allowed to begin surgery.

The intravenous blood samples of the patients in both groups were taken at three times (8 to 10 mL each time), including before tourniquet application (baseline) and 12 and 24 hours after reperfusion. The samples were frozen after centrifugal plasma separation in order to be analyzed at the same time. IL-6 and TNF-*α* were determined by ELISA using Diaclone kits (French) in terms of the manufacturer's instructions. hs-CRP was also quantitated and recorded by immune turbidimetry using ZellBio GmbH kit and Fertin [26] by chemiluminescence using validated standard kits in the laboratory (monobind, USA).

After anesthesia and spinal anesthesia, tourniquet up to 150 mmHg of systolic blood pressure was inflated to prevent blood flow to the limb undergoing surgery (approximately 90 minutes). In order to control the mean arterial blood pressure and normal temperature, we used warm fluid. If arterial pressure is 20% lower than baseline during surgery, it indicates a reduction in blood pressure. A single dose of 5 mg intravenous ephedrine was administered as a bolus dose to regulate the blood pressure and continued to increase by one milligram to return blood pressure to the baseline. When postoperative bleeding occurred, the volume was replaced with salt solution rather than blood products. The duration of closure of tourniquet, the volume of topical anesthetic medicines, and the dose of ephedrine were recorded.

It should be noted that this study was a double-blind one in which the data were collected and recorded by an anesthesia resident who was blinded to the type of intervention. The surgeon and the anesthesiologist were the same in all procedures. The data were analyzed by the researcher who was unaware of the patient groups' classification and results of blood samples related to the type of intervention.

The data were analyzed using SPSS version 22. At first, Kolmogorov-Smirnov test was used to test the normal distribution of the study variables. Then, Chi-square and independent *t*-test were used to compare demographic information and cytokine's level in the two groups, respectively. In order to compare the patterns of inflammatory cytokines during the study period in study groups, repeated measure was used. The significance level was considered *p* < 0.05.

## 3. Results

The mean age and standard deviation of patients in general and spinal anesthesia were 28.65 ± 7.12 and 36.24 ± 14.33 years, respectively (*p*=0.06). The mean of weight in two groups had no significant difference; 88.2% (15 cases) and 82.4% (14 cases) of general and spinal anesthesia were male, respectively. Also, 6% and 17.6% of the patients of general and spinal anesthesia had the history of medicine consumption. Duration of tourniquet closure in 58.8% of the patients of general anesthesia group and 82.4% of patients of spinal anesthesia group was more than 60 minutes; however, this difference was not statistically significant (*p*=0.13). The two groups had similar history of previous disease (*p*=0.61).

**Figure 1:**
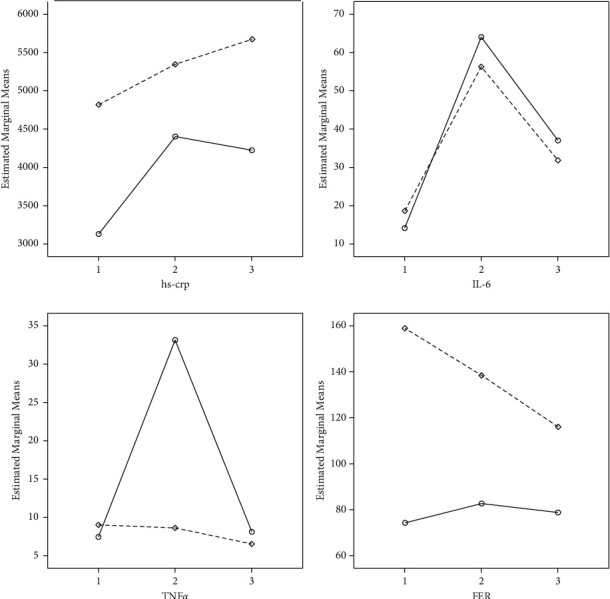
The pattern of changes of hs-CRP, IL-6, TNF-*α*, and ferritin [26] during the study period in two groups. Solid line and dash line show the general and spinal anesthesia, respectively.

According to Table 1, the pattern of changes of inflammatory cytokines has no significant difference between the two studied groups (*p* > 0.05). Furthermore, TNF-*α* and ferritin showed no significant changes in both groups (*p* TNF-*α* = 0.23 and *p* Fer = 0.14). However, hs-CRP and IL-6 had a significant ascending pattern (*p* < 0.001).  Figure 1 shows that the ascending pattern during the study period for hs-CRP and IL-6 has a quadratic function.

The mean changes of IL-6 at different times in the two groups of general and spinal anesthesia showed no statistically significant difference. Mean changes in serum TNF-*α* level before and after 12 hours and before and after 24 hours indicated that no significant difference was found between the two methods of spinal and general anesthesia. No significant difference was observed in the means of serum ferritin level between two groups of spinal and general anesthesia.

## 4. Discussion

Induction of anesthesia by drugs influenced the immune system by affecting immune cells in perioperative period. These effects on immune cells, including T-lymphocytes, B-lymphocytes, macrophages, natural killer cells, and leukocytes, were investigated [27, 28]. The results of the present study showed that the mean serum level of hs-CRP in patients of both studied groups had no significant difference; however, the mean serum hs-CRP level changes during the study period showed significant difference.

Hughes et al. in a study investigated leukocyte activity and changes in inflammatory and coagulation markers caused by ischemia-reperfusion injury following tourniquet closure of the forearm [29]. They studied 10 healthy volunteers who had no history of heart disease at a 24-hour interval. The blood samples of the subjects were taken at baseline, 10 min after ischemia, and 5, 15, 30, and 60 min and 24 h after reperfusion. The results showed that the level of CRP concentration increased during the ischemia compared to baseline, and the maximum peak was at 15 min after reperfusion and reduced by passing 24 h from reperfusion; and at the same time, it was higher than the baseline [29].

Eroğlu et al. compared the effects of general and spinal anesthesia on systemic inflammatory response in patients undergoing total knee joint replacement surgery. The study results showed that, in both groups, CRP levels significantly increased 24 hours after surgery compared to the time of operation; however, no significant difference was found between the two groups [30], which is consistent with the results of the present study.

The results of the present study showed that the mean serum levels of IL-6 significantly increased over time in patients in both general and spinal anesthesia; however, the mean serum levels of IL-6 were not significantly different between the two groups of general and spinal anesthesia. The study results of Eroğlu et al. showed that IL-6 levels were significantly increased in two groups by passing 24 hours from surgery compared to baseline, but no significant difference was found between these two groups [30], which is consistent with the results of the present study. IL-6 and hs-CRP are cytokines and are more sensitive to inflammatory changes in the body and can rapidly respond.

The mean serum TNF-*α* level had no significant changes in both groups and during the study period. Halladin et al. conducted a study entitled “lower limb ischemia and reperfusion injuries in healthy volunteers by inflammatory and oxidative markers” and exposed 10 male volunteers to lower limb ischemia for 20 minutes [31]. They performed muscle biopsies, and blood samples were taken at 5, 15, 30, 60, and 90 minutes after tourniquet closure. The results showed that no significant increase was observed in MDA muscle biopsy after reperfusion. Oxidative plasma levels and pro- and anti-inflammatory parameters (ascorbic acid, dihydroascorbic acid, TNF, IL-1*β*, IL-6, and TNF-R) were not significantly different after reperfusion compared to baseline. They concluded that lower limb ischemia for 20 minutes has not caused ischemia-reperfusion injury in healthy volunteers [31], which is in some parts inconsistent with the results of the present study. In the present study, the closing time of tourniquet was longer than 60 minutes for most of the patients, whereas in a study by Halladin et al. [31] tourniquet closing time was 20 minutes and they concluded that this time was short for producing and/or increasing the inflammatory markers. Probably the difference in the results of the present study and this study may be due to the duration of the tourniquet closure.

The results of various studies have shown that IL-6 levels significantly increased by passing 4 and 24 hours from reperfusion; however, TNF-*α* and IL-1 levels showed no significant increase during this period. In these studies, the closing time of tourniquet was 60 to 120 minutes [8, 31, 32].

Kamat et al. conducted a study on patients undergoing hand surgery. In these patients, the tourniquet was closed in the arm and inflated with 250 mmHg of Mercury, and also the duration of surgery was 116 ± 16 minutes. Accordingly, during surgery, tissue and blood samples were taken from the site of surgery at baseline and 2 and 10 minutes after reperfusion, and inflammatory factors were evaluated. The results showed that cytokines, IL-6, IL-7, IL-17, and TNF-*α* significantly increased at baseline after 10 min [33]. Secretion of proinflammatory (IL-6, Il-8, IL-1, and TNF-*α*) and anti-inflammatory (IL-10) cytokines was influenced by anesthesia. General anesthesia affects the levels of interferon Gamma (IFN-*γ*) and TNF-*α* [28].

The results of an experimental study showed that the highest TNF-*α* response to severe ischemia-induced injury occurred one minute after reperfusion and reached the control level after 10 minutes [34]; and in patients undergoing IL-6, it increased 30 to 60 minutes after surgery and changes in concentration significantly increased after 2 to 4 hours [35].

According to the results of the present study, it can be said that the maximal level of IL-6 in both groups was in 12 hours after surgery. Surgery or trauma induces a stress response, which protects the body. A regional anesthesia blocks neuroendocrine response but could not eliminate the cytokine response [36]. Secretion of IL-1 and TNF-*α* is the initiation of cytokine response, which induces the production of other cytokines, especially IL-6 as the reason of the systemic change [36]. The results of the present study showed that the mean serum fer level in patients of both groups and over time was not significantly different. By performing extensive searches in available databases such as SID, Magiran, Iranmedx, and Pubmed no study was found to have evaluated the level of fer in patients after opening tourniquet and reperfusion; therefore, it was not possible to compare this part of the results of the present study with other studies.

In the current study, the effect of drain use was not investigated due to lower rate of its application that might be useful for investigation in other studies as it increases the chance of inflammation [37]. Using tourniquet reduces intraoperative hemorrhage, allows better visualization of the surgeon, a is bloodless site, and finally reduces the surgery duration [5]. Also, it can be said that, in a healthy person under 50 years of age, tourniquet should not be closed for more than 2 hours [38]. The rate of two hours is reasonable, because after this period of ischemia, progressive venous acidosis will be developed in the site affected by the tourniquet [39]. An ischemic organ reperfusion is essential to prevent irreversible tissue injuries [40]. The injuries caused by reperfusion of skeletal muscles produce a variety of reactive oxygen species (ROS) and proinflammatory cytokines, which not only affect the muscles, but also cause injuries due to reduced blood flow to the organs [32, 41]. The lower limb ischemia-reperfusion injury can cause local injuries and severe abnormalities of the excreting organs including the lungs and kidneys [41]. Moreover, no hip replacement surgeries were performed in the current study.

### 4.1. Limitation

Because spinal anesthesia required patient consent, the emphasis was not on young people due to headaches. So the mean age of spinal anesthesia went up.

## 5. Conclusion

The pattern of hs-CRP, IL-6, TNF-*α*, and fer of the patients in both groups showed similar trend. The significant change of hs-CRP and IL-6 was observed over time. According to the results of the present study, the use of tourniquet leads to inflammation, and the inflammation is similar in both groups. In order to prevent the possible complications, it is suggested that, in the future studies, the amount of bleeding should be measured and compared between two methods. Moreover, the effect of drain use in the knee surgery is worth investigating. Considering the effect of duration of tourniquet closure on changes in inflammatory factors, it is recommended to conduct studies on patients with ischemia for 30 to 35 minutes and also on samples with reperfusion between 4 and 5 hours after reperfusion. Moreover, the use of disposable instrument was suggested in future studies to investigate the inflammatory responses.

## Figures and Tables

**Table 1:** The pattern of changes of inflammatory cytokines during the study period and between groups.

	General anesthesia			Spinal anesthesia			*p* value^*∗*^	*p* value^+^
	Before	12 hours later	24 hours later	Before	12 hours later	24 hours later		
hs-CRP	3132.29 ± 2318.46	4404.53 ± 2102.69	4225.82 ± 2187.66	4822.94 ± 1393.18	5345.41 ± 603.76	5673.65 ± 431.05	<0.001	0.28
IL-6	14.03 ± 24.42	64.01 ± 79.47	37.04 ± 55.09	18.57 ± 29.09	56.28 ± 42.99	31.80 ± 24.18	<0.001	0.58
TNF-*α*	7.42 ± 2.88	33.15 ± 89	8.10 ± 3.53	9.02 ± 1.10	8.61 ± 2.38	6.54 ± 1.80	0.23	0.25
FER	74.29 ± 49.29	82.72 ± 44.74	78.66 ± 33.12	158.94 ± 177.77	138.51 ± 106.81	116.02 ± 85.19	0.14	0.07

^*∗*^ *p* value for the main effect. ^+^*p* value for the interaction effect. Mauchly's *W* for hs-CRP (*p* value) = 0.92 (0.30). Mauchly's *W* for IL-6 (*p* value) = 0.43 (<0.001). Mauchly's *W* for TNF-*α* (*p* value) = 0.004 (<0.001). Mauchly's *W* for FER (*p* value) = 0.17 (<0.001). Significance of the bold value is < 0.05.

## Data Availability

The data used to support the findings of this study are available from the corresponding author upon request.
